# Botanical Description of British Plants

**Published:** 1808-02

**Authors:** 


### *Botanical Description of British Plants.*

[ Continued from our last, pp. 71—78. ]

*T. melilotus officinalis.*

*Ang.* Melilot trefoil. Common melilot. King's clover Hart's clover.

*Gen. Desc.* Flowers mostly forming a head; capsule scarcely longer than the calyx; not opening, but falling off entire, with 1 or 2 seeds.

*Spec. Desc.* *Caps.* in bunches, often 2 seeded, wrinkled, acute. *Stem upright.* *Cal.* 1¼ in length of the bloss; cleft half way down, segm. equal. *Bloss.* bent back, scattered, yellow. *Leafits* of the *lower* leaves oblong, wedge-shaped; of the *upper*, oval, sharply serrated, toothed.

oothed. *Bunches* long. *Corn fields, meadows, way sides, ditch banks.*—Bl. June, July.

*Use.* It has generally been esteemed *emollient* and *digestive;* used in fomentations and cataplasms, particularly in the plaister employed for dressing blisters; but it is now laid aside, as its quality is found to be rather *acrid* and irritating, than emollient or resolvent. It communicates a loathsome flavor to wheat and other grain, and makes it unfit for bread.—*Lightfoot.* A distilled water from the flowers possesses little odour in itself, but improves the flavor of other substances. The plant is more fragrant when dry than when green.—Horses are extremely fond of it; cows, sheep, goats and swine eat it.—*Withering.*

13. Trifolium. *T. pratense.*

*Ang.* Purple trefoil. Honeysuckle trefoil. Cow clover. Clover grass.

*Gen. Desc.* *As above.*

*Spec. Desc.* *Spikes* crowded. *Calyx.* with 4 of the teeth equal. *Stipula* awned. *Stems* ascending. *Bloss.* unequal, of 1 pet. tube long, standard longer than the wings and keel, blunt, notched, purple, sometimes white. *Meadows.* Bl. May, Sept.

*Use.* The heads are used in Sweden to dye woollens green; with alum they afford a light, and with copperas a dark green.—*Withering.*

14. Lotus. *L. corniculatus.*

*Ang.* Bird's-foot clover.

*Gen. Desc.* Cal. tubular. Wings converging lengthways and upwards. Legumens straight, generally with cells.

*Spec. Desc.* *Heads* of the flowers flatted at the top, with a single sitting leaf at the base. *Stem* herbaceous, trailing. *Legum.* cylind. expanding, ending in a straight point. *Cal.* fringed. *Bloss.* sweet-scented; before opening, bloody red without, yellow green within; when expanded, full yellow; *standard* bent back; wings oblong, egg-shaped. *Pet.* equal, on separate claws. *Meadows, heaths, road sides.* Bl. June, August.

*Use.* The flowers become greenish when dried, in which respect they resemble the flowers of the plants which produce indigo. In Hertfordshire it is cultivated as pasturage for sheep; it is strongly recommended by Mr. Anderson; in moist meadows it grows to a great height, and makes extremely good hay; there is no doubt it might be cultivated to advantage.—Horses, cows, and goats eat it; sheep and swine are not fond of it.—*Withering.*

### CLASS XVIII. POLYADELPHIA.

### Polyadelphia. Polyandria.

1. HYPERICUM. *H. perforatum. H. vulgare.*

*Ang.* St. John's wort. Perforated St. John's wort.

*Gen. Desc.* Cal. five div. Petal 5. Filaments numerous, united at the base into 3 or 5 sets. Capsule 1, 3, or 5 celled, many-seeded. Styles 1, 3, or 5.

*Spec. Desc.* *Stem* two-edged, upright, nearly cylindrical, beset with black dots. *Leaves* blunt, in cross pairs, oblong, with pellucid dots, with 5 or 7 semi-transparent lines, and with black dots near the edges underneath. *Fruit-st.* axillary. *Pet.* ribbed. Set near the edges with dark purple glands, one side entire, the other serrated. *Stem.* 30 or more. *Anth.* with a globular black gland at the top between the lobes. *Germen* egg shaped. *Styles* yellow. *Sum.* sometimes crimson.—*Thickets, hedges, dry banks.* BL. July.

*Use.* It has a bitterish sub-astringent taste, and sweetish smell. It was in great repute with the ancients, who prescribed it in hysteria, hypochondriasis, and mania: they also imagined that it had the peculiar power of curing demoniacs, and thence it obtained the name of Fuga dæmonum: it was also recommended internally for wounds, bruises, ulcers, hæmoptysis, bloody urine, gravel, dysentery, agues, worms; and outwardly as an anoydone, and as a discutient and detergent. It is now however rarely used, and has been omitted in the Edinb. Mat. Med.: in that of the London Coll. the flowers only are directed to be used, as containing the greatest proportion of the resinous oily matter in which the medical efficacy of the plant is supposed to reside: the dark puncta of the petals and capsules afford this essential oil, which is contained in minute vesicles, and gives a red colour to rectified spirit, and to expressed oils: it gives a good dye to wool.—*Woodville.* The use of this plant, though it has long held a place in the Materia Medica, is very much undetermined. The leaves given in substance, are said to destroy worms. The flowers tinge spirits and oils of a fine purple colour, derived probably from the little glands upon the anthers, and upon the edges of the petals. The semi-transparent dots upon the leaves are the receptacles of an essential oil.—*Withering.* An oil or tincture of the flowers is esteemed a good vulnerary: the expressed juice or infusion of the same is reckoned useful to destroy worms, to resolve coagulated blood, and to promote urine. The

Scotch

Scotch imagine that ropy milk is cured by putting this plant into it, and milking on it a-fresh. The Swedes, with the flowers, give a purple tinge to their spirituous liquors.—*Lightfoot.* This species is distinguished at one view by the little black gland on the anthers.—*Curtis.* Cows, goats, and sheep eat it; horses and swine refuse it.

2. HYPERICUM.* *H. androsæmum.*

*Ang.* Tutsan. Park-leaves.

*Gen. Desc.* As above.

*Spec. Desc.* *Stem* shrub-like, two-edged. *Fruit* like a berry. *Leaves* opposite, sitting, smooth, entire, egg-shaped; v. large at the base, smaller as they ascend. *Cal.* sym. unequal. *Berry* black. *Fruit-st.* cylind. smooth. *Flowers* terminating, 4 together. *Pet.* yellow, concave, scored, unequal at the end from a hollow in the margin at one side. *Woods, moist hedges.* BL. July, Sept.

*Use.* This is a good vulnerary; the leaves readily healing any fresh wounds: and thence it has derived the name of Tutsan from the French *Tout-sain,* or All-heal.—*Lightfoot.*

### CLASS XIX. SYNGENESIA.

### Syngenesia. Æqualis.

1. TRAGOPOGON. *T. pratense.*

*Ang.* Yellow goat's-beard. Go to bed at Noon.

*Gen. Desc.* Receptacle naked. Calyx simple. Down feathered.

*Spec. Desc.* *Cal.* as long as the rays of the bloss. *Leaves* entire, straight, long and narrow, tapering. *Fruit-st.* cylind. *Bloss.* yellow, expanding 3 A. M. and closing about 9 or 10 A. M. unless the sky be cloudy. *Anth.* purple. *Pollen* yellow. *Seeds* crooked. *Whole plant* smooth, stiff, strong, upright. *Meadows and pastures.* BL. June.

*Use.* The roots, cut before the stems shoot up, and boiled as asparagus, have the same flavour, and are nearly as nutritious.—Swine devour this plant greedily: horses, cows and sheep eat it; goats are not fond of it.—*Withering.*

2. TRAGOPOGON. *T. porrifolium.*

*Ang.* Purple goat's-beard. Salsafy.

*Gen. Desc.* As above.

*Spec. Desc.* *Cal.* one-third longer than the rays of the blossom. *Leaves* entire, stiff and straight, short. *Fruit-st.* thickening upwards. *Florets* very narrow, topped. *Bloss.* purple.—*Meadows and pastures.* BL. May.

*Use.* The roots are esculent, and are cultivated in the garden, by the name of Salsafie.—*Withering.*

3. Picris. *P. echioides.*

*Ang.* Common ox-tongue. Lang de bouf.

*Gen. Desc.* Recept. naked. Calyx double. Down feathered. Seeds with transverse furrows

*Spec. Desc.* *Outer cal.* five-leaved, larger than the inner; the inner awned. Down the length of the in. cal. on a foot-st. 3 or 4 lines long. *Root-leaves* oval, scolloped, stiff, with numerous watery protuberances set with short hairs. *Stem* firm, cylind. scored, purplish, branched, with scattered stiff thorn-like hairs. *Leaves* heart-shaped, waved, set with sharp hairs. *Flowers* single, on fruit-st. yellow, expanding at 4 or 5 a. m. open till 9 p. m.—*Borders of corn-fields.* Bl. July, Aug.

*Use.* This plant when young is an agreeable pot-herb: the juice is milky, but not too acrid.—*Withering.* The seeds are shining, and when viewed through a glass, extremely beautiful.

4. Lactuca. *L. virosa.*

*Ang.* Wild lettuce. Strong scented lettuce.

*Gen. Desc.* Recept. naked. Cal. tiled, cylindrical, the scales membranaceous at the edge. Down hair-like, on a pedicle.

*Spec. Desc.* *Leaves* horizontal, toothed; mid-rib prickly on the back; arrow-shaped, sitting, embracing, either entire or wing-cleft. *Stem* 2 to 4 feet high, prickly below. *Flowering branches* expanding. *Cal.* Scales unequal, spear-shaped. *Bloss.* numerous, yellow, open at 7, close at 10 a. m. *Seeds* black, rough·—*Ditch-banks.* Bl. July, Aug.

*Use.* The juice smells like opium, is milky, acrid, and bitter. Dr. Collin relates 24 cases of dropsy, of which 23 were cured by taking the extract prepared from the expressed juice, in doses from 18 gr. to 3 drachms in the 24 hours. It commonly proves laxative, promotes urine and gentle sweats, and removes thirst. It must be prepared when the plant is in flower.—*Withering.* *Woodville.*

5. Leontodon. *L. officinalis.—Densleonis.* *Taraxacum.*

*Ang.* Dandelion. Piss-a-bed.

*Gen. Desc.* Recept. naked. Cal. tiled; miner scales parallel, equal. Down hair-like.

*Spec. Desc.* *Down* on a pedicle. *Outer scales of the al.* entire, reflected, smooth. *Leaves* smooth, notched, and and acutely toothed, varying from wing-cleft in a dry situation to nearly entire in a moist one. *Seeds* flat, scored, prickly upwards. *Bloss.* yellow, expanding at 5 or 6 A. M. closing early P. M.—*Meadows, road-sides, ditch-banks, &c.* BL. April, Sept.

N. B. *This seems to be the same species as that denominated by Dr. Woodville, Bergius, Haller, &c. &c. L. taraxacum, though the latter is by Dr. Withering enumerated as a distinct species.*

*Use.* Dandelion is considered as the most active and efficacious of the lactescent plants: the expressed juice is bitter and somewhat acrid; but the root is still bitterer, and possesses more medicinal power than any other part of the plant. This plant has been long in repute as a mild detergent and aperient, and its diuretic effects may be inferred from the vulgar name it bears in most European languages, as Piss-a-bed, Piss-au-lit, &c. which quality seems to be particularly efficacious during sleep.—*Ray. Murray* speaks of the root as resolving viscid humours, and healing eruptions, &c. *Bergius* recommends its use in obstructions of the liver, hypochondriasis and jaundice: in the first of these diseases its successful use was confirmed by his own experience. See *Berg.* M. M. t. ii. p. 649. *De Haen* mentions an instance of its good effects in the same complaint; and various proofs are recorded by different authors of its beneficial use in jaundice, dropsy, pulmonic tubercles, and some cutaneous disorders. The leaves, roots, flower-stalks and juice have all been separately employed for medical purposes, and seem to differ rather in degree of strength than in any essential property: the expressed juice, or a strong decoction of the roots, have most commonly been prescribed from 1 oz. to 4, two or three times a day. The plant should be always used fresh; even extracts prepared from it appear to lose much of their power by keeping.—*Woodville. Boerhaave* had a great opinion of the utility of this and other lactescent plants in visceral obstructions; the expressed juice has been given to the quantity of 4 oz. three or four times a day. Early in the spring, while the leaves are yet white and hardly unfolded, they taste like endive, and make an excellent addition to those plants eaten in sallads at that time of the year. The French eat the roots and the blanched leaves with bread and butter. Children who eat it in the evening are apt to experience its diuretic effects, and thence its vulgar name. In Minorca, when a swarm of locusts had once destroyed the harvest, this plant afforded forded subsistence to many of the inhabitants.—*Withering.* At Gottingen, it is a practice with the poorer inhabitants to use the roots of this plant, roasted, as a substitute for coffee; and an infusion, prepared in this way, can scarcely be distinguished from that of the coffee-berry.—*Murray's Apparat. p.* 107. These flowers seem to possess a certain degree of sensibility, for under the powerful influence of the sun in a summer's morning, an evident motion of the flowerets may be discovered.—*Dr. Hope's M.S. Lect.*—Swine devour it greedily; goats eat it; cows and sheep are not fond of it; horses refuse it. Small birds are fond of the seeds.

( To be continued. )

